# Phospho-^T356^RB1 predicts survival in HPV-negative squamous cell carcinoma of the head and neck

**DOI:** 10.18632/oncotarget.4321

**Published:** 2015-05-29

**Authors:** Tim N. Beck, John Kaczmar, Elizabeth Handorf, Anna Nikonova, Cara Dubyk, Suraj Peri, Miriam Lango, John A. Ridge, Ilya G. Serebriiskii, Barbara Burtness, Erica A. Golemis, Ranee Mehra

**Affiliations:** ^1^ Molecular Therapeutics, Fox Chase Cancer Center, Philadelphia, PA, USA; ^2^ Medical Oncology, Fox Chase Cancer Center, Philadelphia, PA, USA; ^3^ Surgical Oncology, Fox Chase Cancer Center, Philadelphia, PA, USA; ^4^ Molecular and Cell Biology and Genetics, Drexel University College of Medicine, Philadelphia, PA, USA; ^5^ Department of Internal Medicine, Yale University School of Medicine and Yale Cancer Center, New Haven, CT, USA; ^6^ Department of Biochemistry, Kazan Federal University, Kazan, Russia

**Keywords:** biomarkers, RB1, CDK4/6, E2F, head and neck cancer

## Abstract

Locally advanced squamous cell carcinoma of the head and neck (SCCHN) that is not associated with human papillomavirus (HPV) has a poor prognosis in contrast to HPV-positive disease. To better understand the importance of RB1 activity in HPV-negative SCCHN, we investigated the prognostic value of inhibitory CDK4/6 phosphorylation of RB1 on threonine 356 (T^356^) in archival HPV-negative tumor specimens from patients who underwent surgical resection and adjuvant radiation. We benchmarked p^T356^RB1 to total RB1, Ki67, p^T202/Y204^ERK1/2, and TP53, as quantified by automatic quantitative analysis (AQUA), and correlated protein expression with tumor stage and grade. High expression of p^T356^RB1 but not total RB1 predicted reduced overall survival (OS; *P* = 0.0295), indicating the potential relevance of post-translational phosphorylation. Paired analysis of The Cancer Genome Atlas (TCGA) data for regulators of this RB1 phosphorylation identified loss or truncating mutation of negative regulator CDKN2A (p16) and elevated expression of the CDK4/6 activator CCND1 (cyclin D) as also predicting poor survival. Given that CDK4/6 inhibitors have been most effective in the context of functional RB1 and low expression or deletion of p16 in other tumor types, these data suggest such agents may merit evaluation in HPV-negative SCCHN, specifically in cases associated with high p^T356^RB1.

## INTRODUCTION

Squamous cell carcinoma of the head and neck (SCCHN) is the sixth most common cancer globally [1]. In spite of advances in the development of therapies targeting proteins such as EGFR (epidermal growth factor receptor), other ErbB family members [2, 3] or c-MET [4], which are highly expressed in many SCCHN tumors, the mainstays of treatment for SCCHN remain surgery, cytotoxic chemotherapy, and radiation [5]. In recent decades, the emergence of SCCHN of the oropharynx related to transforming oncogenic HPV infection has been recognized. [6]. Three-year survival rates for locally advanced HPV-negative SCCHN compared to HPV-positive cases are significantly reduced (57.1% *versus* 82.4%; [6]). Given the poor prognosis for locally advanced HPV-negative SCCHN, the identification of prognostic markers that might inform the utilization of targeted therapies, optimize current treatment approaches, and match patients to appropriate clinical trials is necessary.

In HPV-positive disease, the virally encoded oncoproteins E6 and E7 accelerate degradation of the TP53 and RB1 tumor suppressors, respectively [7], and are essential for tumorigenesis. In HPV-negative SCCHN, the impact of RB1 levels and phosphorylation status has been less studied. RB1 is highly regulated and critical for cell cycle progression and tumor suppression [8-12]. Primary regulation of RB1 is accomplished via phosphorylation by cyclin-dependent kinases (CDKs), specifically CDK2, CDK4 and CDK6, which in the case of CDK4 and CDK6, complex with cyclin D (CDK2 complexes with cyclins E and A) [8]. Phosphorylation of RB1 is influenced by p16, encoded by *CDKN2A*, which disrupts CDK4 binding to cyclin D (CCND1) [13, 14]. CDKN2A is often lost in HPV-negative SCCHN, based on LOH of the locus 9p21-22, or by epigenetic silencing [15-18]. CDK4/CDK6-cyclin D dependent phosphorylation of RB1 on tyrosine residue 356 (pT^356^) [19], a residue located in the interdomain linker separating the RB1 N-terminal domain (RBN) region and the pocket domain, was recently defined as disrupting the interaction of RB1 with E2F1 to induce cell cycle progression, and indicates functional inactivation of the protein (CDK4/CDK6-cyclin D definitively phosphorylates RB1 at T^356^, while it is less clear if CDK2 also phosphorylates RB1 at T^356^ [19-23]). This finding suggested that measurement of phospho-T^356^RB1 might strongly predict RB1 activity and given the crucial role of RB1, p^T356^RB1 levels may be informative in terms of patient outcome for cases of HPV-negative SCCHN.

From a therapeutic standpoint, recent FDA approval of the CDK4/6 inhibitor palbociclib for the treatment of ER+/HER− breast cancer [24] emphasizes current interest in the CDK4/CDK6-cyclin D to RB1 signaling axis [25]. In addition to breast cancer, melanoma, non-small cell lung cancers, lymphomas, ovarian cancers, and liposarcomas are currently being treated with CDK4/6 inhibitors in clinical trials [25-30], with two clinical trials in progress for SCCHN [NCT00824343; NCT00899054] using the CDK inhibitor P276-00 (predominantly targets CDK4, CDK2 and CDK9; [31]). It is generally accepted that for CDK4/6 inhibitors to be active, functional RB1 is required [25, 27, 30]. In the context of CDK4/6 inhibition, RB1 remains de-phosphorylated and bound to E2F1 [22, 25, 28, 30]. p16 status has been proposed as a response predictive biomarker for CDK4/6 inhibitors, based on the suppressive role of p16 on CDK4/6 and the feedback loop that results in high levels of p16 when RB1 expression is suppressed [8, 30]. To date, in clinical or preclinical studies of melanoma [32], ovarian cancer [33], pancreatic cancer [34] and glioblastoma [35], low p16 expression has been associated with CDK4/6 inhibitor sensitivity, whereas in breast cancer, p16 levels were not predictive, leaving its overall value as a response biomarker unclear at present [24, 36]. Further, in the specific case of SCCHN, p16 may be non-optimal as a biomarker in HPV-negative disease, given that high levels of p16 often are used in screening for HPV-positive, virally dependent cancers [37-39].

In this study, we have evaluated phospho-T^356^RB1 as a possible prognostic indicator for HPV-negative SCCHN. We have also correlated expression of phospho-T^356^RB1 with activation of ERK1/2 (phospho-T^202^/Y^204^), which is typically associated with active cell cycle [40]; with the proliferative indicator Ki-67; and with other protein biomarkers. Finally, we examined the relationship between phospho-T^356^RB1 and known clinical outcome markers including T classification, N classification, and tumor grade. These data for the first time indicate a strong predictive function of p^T356^RB1 for prognosis in SCCHN, and a striking relation between p^T356^RB1, p^T202/Y204^ERK1/2, and Ki-67 expression.

## RESULTS

### Patient characteristics and AQUA analysis

TMAs were constructed using tissue obtained from 94 HPV-negative patients who underwent surgery (Table 1). Sub-sites were predominantly oral cavity (44%), oral tongue (22%) and glottis (17%; Table 1). As is typical for HPV-negative SCCHN, high T-stage and high N-stage significantly predicted poor survival outcomes (*P* = 0.043 and *P* = 0.045 respectively), while tumor grade did not (*P* = 0.160; Supplementary Figure S1). T1 or T2 had a median survival of 124.0 months and N0 or N1 of 88.1 months. The median survivals for high T- and N-stage were 47.0 months and 24.5 months, respectively.

**Table 1:** Patient characteristics for the analyzed TMA

	All (S/SRT)		Surgery + Radiation (SRT)	
**Gender**				
Female	35	37%	24	37%
Male	59	63%	45	65%
**Age at diagnosis**				
Mean	62.8		61.8	
Min	25		25	
Max	86		86	
SD	12.9		13.3	
**Primary site**				
Glottis	16	17%	14	20%
Hypopharynx	3	3%	2	3%
Oral cavity	41	44%	31	45%
Oral tongue	21	22%	13	19%
Oropharynx	8	9%	5	7%
other	5	5%	4	6%
**Overall stage**				
1	14	15%	3	4%
2	8	9%	1	1%
3	14	15%	10	14%
4	58	62%	55	80%
**T stage**				
1	19	20%	7	10%
2	24	26%	16	23%
3	16	17%	14	20%
4	35	37%	32	46%
**N stage**				
0	44	47%	24	35%
1	12	13%	9	13%
2	38	40%	36	52%
**M stage**				
0	93	99%	68	99%
X	1	1%	1	1%
**Grade**				
Well diff.	9	10%	5	7%
Moderately diff.	55	59%	40	58%
Poor/Undiff.	30	32%	24	35%
**Positive staining**				
TB53	94		69	
p^T202^RB1	79		61	
RB1	55		38	
Ki67	89		66	
p^T202^/^Y204^ERK1/2	85		64	

**Figure 1:**
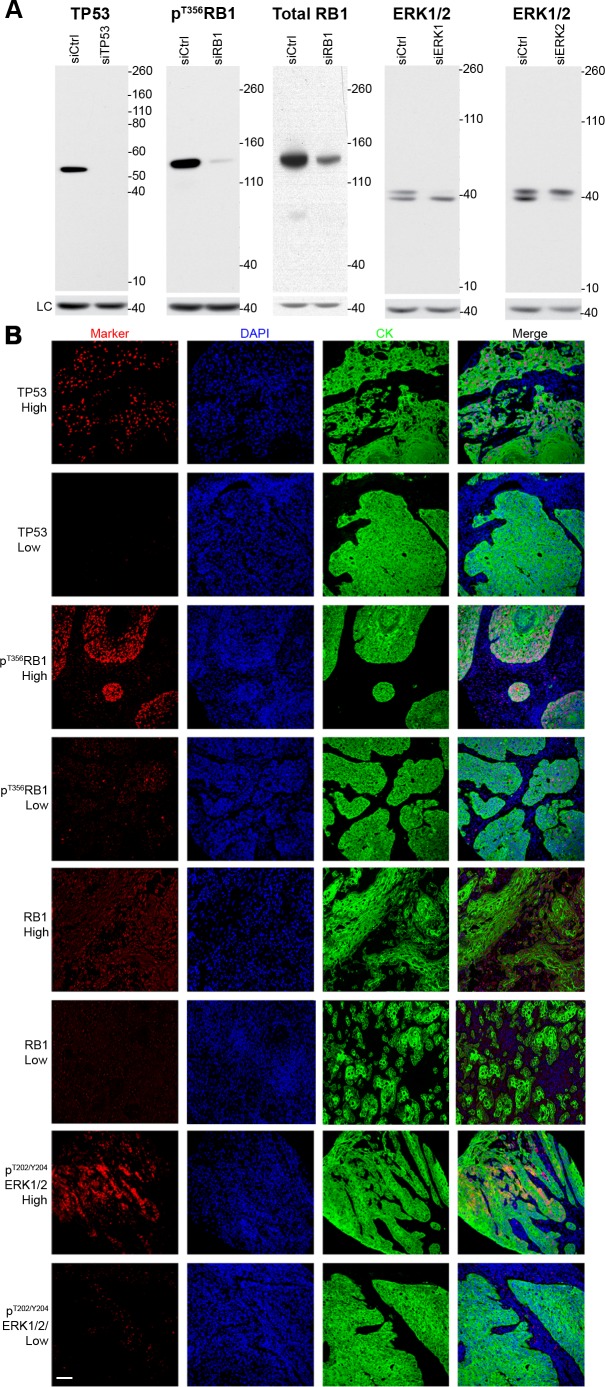
Validation of antibodies and immunofluorescence microscopy **A.** Western blots for the relevant protein markers in the presence or absence of siRNA, **B.** representative high and low staining immunofluorescent microscopy images for each marker. LC = loading control (β-actin), DAPI = nuclear stain, CK = cytokeratin (epithelial tumor stain).

For each protein of interest (p^T356^RB1, RB1, TP53, p^T202/Y204^ERK1/2, and Ki-67), antibodies for AQUA-based assays were validated as specific for the target protein (Figure 1A). In staining of primary tumor tissue, indicated by co-staining with cytokeratin to visualize epithelial tissue [41, 42], comparison of low *versus* high staining tissue for each marker indicated a robust dynamic range (Figure 1B). As specimens were collected over an extended period, we also performed secondary analysis to exclude the possibility that degradation of antibody epitopes over time influenced signal intensity and survival outcomes (Supplementary Figure S2 and Table S1), as has previously been reported [43, 44]. This indicated a low correlation index between age and signal intensity, indicating stability of antigens including phosphoantigens. Based on assessment by a pathologist, to ensure tissue quality and proper staining for each sample, the number of informative samples for each individual marker ranged from 55 to 94 cases (Table 1). We analyzed patients in two different categories: all patients (*n* = 94; includes patients who underwent surgery alone and patients who received surgery plus radiation; referred to as S/SRT), and only patients treated with surgery plus radiation (SRT; *n* = 69; Table 1).

### High p^T356^RB1 strongly predicts reduced overall survival

**Figure 2:**
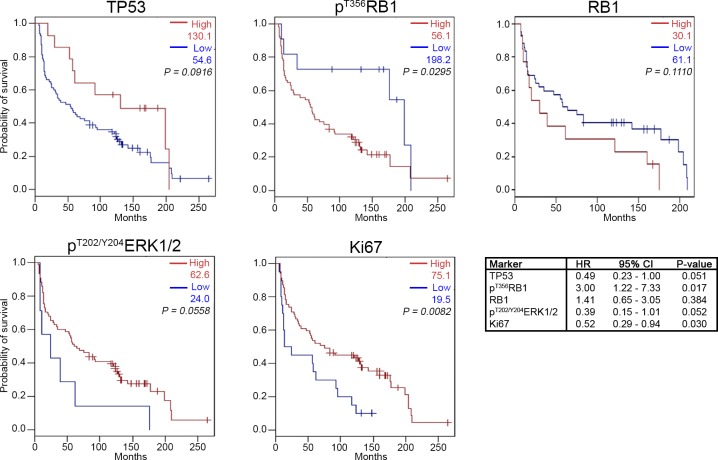
Kaplan-Meier survival analysis for high and low expression levels of TP53, RB1, p^T356^RB1, p^T202/Y204^ERK1/2, and Ki67 Patients treated with surgery only and patients treated with surgery and radiation therapy were included. OS = overall survival; HR = hazard ratio; CI = confidence interval. See Supplementary Table S1 for additional details regarding the HR.

We first considered individual markers as predictors of overall survival (OS; Figure 2 and Supplementary Figure S3). High p^T356^RB1 signal, indicating inactivated RB1 protein [22], strongly predicted reduced OS in both treatment populations, with the effect most apparent in the SRT population (SRT, 27.0 *versus* 198.0 months, *P* = 0.0078; and S/SRT, 56.1 *versus* 198.2 months, *P* = 0.0295). Total RB1 levels did not correlate with survival probability (*P* = 0.1110). As a benchmark, higher proliferation-associated Ki-67 staining strongly predicted extended OS in the S/SRT populations (75.1 *versus* 19.5 months, *P* = 0.0082), but was not significant in the smaller SRT population (43.0 *versus* 19.5 months, *P* = 0.07787), although the general trend was the same. Similarly, higher p^T202/Y204^ERK1/2 staining was near significant for improved survival in the S/SRT population (*P* = 0.0558) but not in the SRT population (*P* = 0.1477).

For contrast, we also analyzed TP53. In SCCHN, disruptive mutations in TP53 are associated with a hazard for death of 1.7 in resectable disease [45, 46]. TP53 mutations in SCCHN include frameshifts, nonsense mutations and deletions (343 out of 2022 tumors (17%); Catalogue Of Somatic Mutations In Cancer (COSMIC) [47]), and also commonly include missense mutations (870 out of 2022 tumors (43%); COSMIC [47]) that are sometimes of unknown significance, and may result in partially functional proteins [48]. Thus, retained expression of TP53 may be of functional significance, or correlate with inactivated RB1. In the TMAs examined, we observed a non-statistically significant trend towards extended OS for higher levels of TP53 (S/SRT, 130.1 *versus* 54.6 months, *P* = 0.0916, and SRT, 130.1 *versus* 31.5 months, *P* = 0.1011; Figure 2 & Supplementary Figure S3).

We additionally performed multivariate analyses using Cox proportional hazards regression to adjust for T-stage, N-stage, grade, gender, patient age, and age of specimen (Figure 2 and Supplementary Table S1). After correction, the hazard ratio (HR) for high p^T356^RB1 was 3.00 (adjusted *P* = 0.017; Figure 2). The second marker with a significant adjusted *P*-value was Ki67 (*P* = 0.030): the corresponding HR was 0.52. RB1, TP53 and p^T202/Y204^ERK1 did not have significant adjusted *P*-values (*P* = 0.384; *P* = 0.051 and *P =* 0.052, respectively).

### High p^T356^RB1 correlates with elevated p^T202^/^Y204^ERK1/2 and Ki67

**Figure 3:**
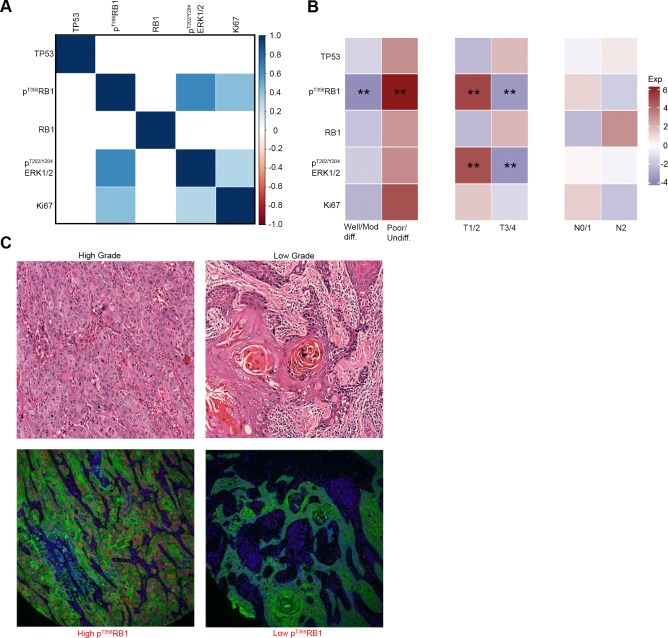
Expression correlation between markers and between markers and tumor stage and grade **A.** Statistically significant correlations between marker expression levels, increasing saturation of blue indicates higher correlation, correlations with *P* > 0.05 are suppressed, **B.** correlations between marker expression levels and tumor grade, T-stage and N-stage, blue squares indicate low marker expression levels and red squares indicate high maker expression levels, **C.** hematoxylin and eosin (H&E) stained samples for high and low grade tumors with the corresponding p^T356^RB1 staining. (**) *P* < 0.05.

We next considered the possibility that significant correlations might be observed among the expression of the five assessed biomarkers, reflecting involvement in common cellular processes. In order to specifically analyze the relationship between p^T356^RB1 and total RB1, only samples with available RB1 staining (*n* = 55) were considered. The most striking correlations seen were noted between high expression of p^T356^RB1 and p^T202/Y204^ERK1/2 (*ρ =* 0.64*; P* < 0.0001) and between p^T356^RB1 and Ki67 (*ρ =* 0.42*; P* = 0.002). Additionally, high Ki67 expression correlated with high p^T202/Y204^ERK1/2, although to a lesser degree (*ρ =* 0.29*; P* = 0.039). No significant correlation between total RB1 and p^T356^RB1 was detected, and TP53 expression did not correlate with any of the other biomarkers examined (Figure 3A). In correlating markers to pathological properties of tumors (Figures 3B and 3C), high expression of p^T202/Y204^ERK1/2 (*P* = 0.047) and p^T356^RB1 (*P* = 0.010) in each case correlated with low T-stage (T1/2; Figure 3B). High p^T356^RB1 levels also strongly correlated with poorly differentiated or undifferentiated disease (*P* = 0.005; Figure 3B and 3C).

### Genomic and transcriptomic analysis supports relevance of high p^T356^RB1 as an indicator of poor outcome

**Figure 4:**
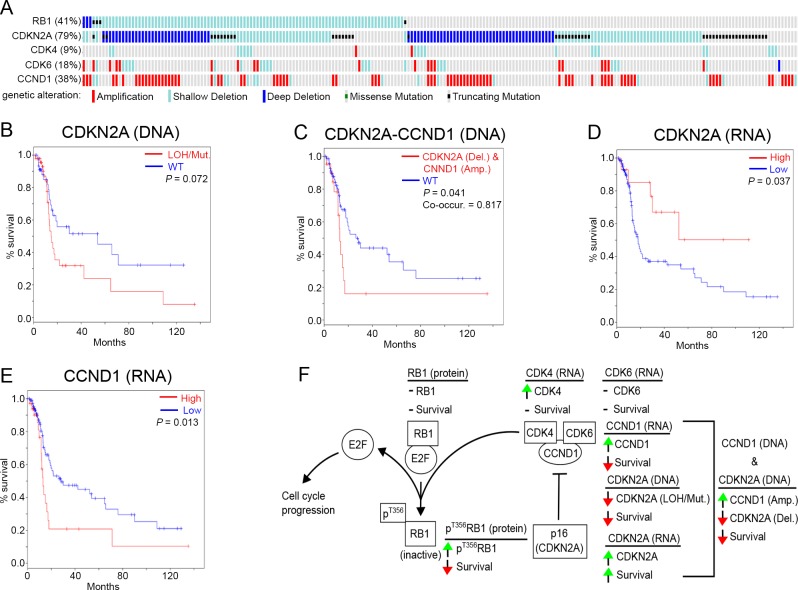
Genomic and transcriptomic analysis of RB1, CDK4, CDK6, CDKN2A (p16) and CCND1 **A.** Genomic alterations of RB1, CDKN2A (p16), CDK4, CDK6 in 243 HPV negative SSCHN TCGA specimens (only tumors with changes affecting at least one of these genes are shown), **B.** Kaplan-Meier survival analysis of CDKN2A with deletions (Del.) compared to WT, **C.** Kaplan-Meier survival analysis for cases of simultaneous CCND1 amplification (Amp.) and CDKN2A homozygous deletion (Del.), **D.** Kaplan-Meier survival analysis of high versus low RNA expression for CDKN2A (p16) and **E.** CCND1. **F.** Summary of RB1-pathway alterations and the survival impact thereof. See Supplementary Figure S4 for additional data. Shallow Deletion = LOH = loss of heterozygosity; Deep Deletion = homozygous deletion; Mut. = mutation; WT = wild type; green arrow = increase; red arrow = decrease; horizontal line = no change; co-occur. = trend towards co-occurrence.

If high p^T356^RB1 predicts poor outcome, then increased activity of CDK4/6 should also predict poor survival, and may be detectable by genomic biomarkers. Increased CDK4/6 activity can be caused by loss of expression or function of the kinase inhibitor CDKN2A (p16) or elevated mRNA expression and/or activating mutation of CDK4, CDK6, and CCND1 (cyclin D1). Inversely, reduced activity of CDK4/6 might be linked to overexpression of CDKN2A. To extend our analysis, we examined the expression and mutational status of RB1 and functionally interacting proteins, including CDKN2A (p16), CDK4, CDK6 and CCND1 in a TCGA dataset of 243 cases of HPV-negative SCCHN. The dominant detected genetic alteration for CCND1 and CDK6 was gene amplification, with most amplifications affecting CCND1 (76/243); while most alterations affecting CDKN2A resulted in shallow deletions reflecting loss of heterozygosity (LOH), deep deletions associated with homozygous deletions, and truncating mutations (192/243; Figure 4A). Protein-damaging RB1 mutations or deletions were detected in only 7 out of 243 specimens, without any discernible effect on survival (Supplementary Figure S4A). A high number of shallow deletions were also detected for RB1, again without indication of any impact on survival (Supplementary Figure S4A). We next investigated whether loss of CDKN2A or RB1 is associated with significant survival implications and found that patients with mutations or LOH of the CDKN2A locus (78/243 tumors) had a strong tendency towards a reduction in survival (*P* = 0.072; Figure 4B). Amplification of CCND1 was independently not predictive of response (*P* = 0.75; Supplementary Figure S4B); however, further analysis indicated significant co-occurrence between CCND1 amplification and homozygous deletion of CDKN2A (co-occurrence ratio: 0.817; *P* = 0.041), with significant impact on survival (Figure 4C).

We also analyzed changes in mRNA expression. The most commonly overexpressed genes in the group were CDKN2A (44/243 with a 2-fold and 28/243 cases with a >3-fold expression increase) and CCND1 (29/243 with a 2-fold expression increase), with lower levels of overexpression seen for RB1, CDK4 and CDK6 (Supplementary Figure S4C and S4D). Overexpression (z > 3-fold) of CDKN2A was robustly associated with increased survival (Figure 4D). In contrast, overexpression of CCND1 (z > 2-fold) correlated with reduced survival (*P* = 0.013; Figure 4E). Finally, CDK6 overexpression did not significantly change survival (Supplementary Figure S4E) and significant overexpression of CDK4 and RB1 was not present in sufficient cases for meaningful analysis (Supplementary Figure S4C and S4D).

## DISCUSSION

While genomic profiling of tumors yields insight into tumor subclasses with distinct prognoses [49], identification of protein expression patterns is useful in cases where significant biological effects are associated with post-translational modification of functionally important proteins [41, 50]. RB1 activity has been much investigated in HPV-positive SCCHN and cervical cancer, but less so in HPV-negative disease. Our research for the first time highlights the potential clinical value specifically of p^T356^RB1 as a biomarker in HPV-negative SCCHN. Patients harboring tumors with low levels of p^T356^RB1 had a median survival nearly four times the median survival for patients with high p^T356^RB1 expression (198.2 *versus* 56.1 months; *P* = 0.0295) when treated with surgery or surgery plus radiation (Figure 2). The difference was even more pronounced when only patients who received surgery plus radiation, a population with more adverse clinical risk factors, were considered (198.0 months *versus* 27.0 months; *P* = 0.0078; Supplementary Figure S2). The observed differences were mediated at the post-translational level, as expression levels of total RB1 did not correlate with survival (Figure 2 and 3). These findings can potentially be explained by the observation that alterations in the RB1-pathway are associated with increased sensitivity to ionizing radiation [51] and furthermore suggest that phosphorylation status of RB1 is critical. We further found that low levels of p^T356^RB1 correlated with low-grade disease (Figure 3B and 3C).

As RB1 is infrequently mutated in HPV-negative SCCHN (Figure 4A; [49]), these data suggest that phosphorylation of T^356^ may serve an important role in functionally inactivating tumor suppression in aggressive disease. The discrepancy between total RB1 and p^T356^RB1 in terms of survival probability highlights the importance of interrogating post-translational events that control protein activity. TCGA databases currently do not include information on T^356^RB1 and, although proteomics-based analyses are in progress, no systematically validated data is currently available, underscoring the value of complementary analysis of protein expression on TMAs. It is possible that on a biological level, a low level of p^T356^RB1 is very different from a complete loss of RB1, based on interactions of RB1 with partners other than E2F1. While multiple potential factors can result in low levels of T^356^ phosphorylation, all, as far as is known, are linked to CDK4/6-CCND1, which are necessary to phosphorylate this residue of RB1 and are negatively regulated by CDKN2A [19, 22, 23, 51-53]. In this study, TCGA data analysis of RB1, CCND1, CDK4, CDK6, and CDKN2A corroborated the potential value of considering inhibition of T^356^RB1 phosphorylation status. Most significantly, overexpression of CCND1 (cyclin D) mRNA, or loss of CDKN2A expression or function, particularly in conjunction with CCND1 overexpression, significantly correlated with worse outcome (Figure 4B-4E). Of note, the CCND1 and CDKN2A loci show some of the highest frequencies of amplification and deletion rates seen in SCCHN and several other cancer types [30]; this further supports the idea that it may be important to restrain RB1 function post-translationally. Larger cohort studies are required to investigate and confirm these ideas.

As summarized in Figure 4F, our data are compatible with a model in which control of RB1 activity is regulated by inhibitory CDK4/6 phosphorylation that varies in tumors based on regulation at the level of CCND1, CDKN2A, or both. Our findings also align with the recognition that oncogenic signaling changes can target multiple points in tumor pathways rather than single critical genes, such as activation of the RAS pathway by mutation or overexpression of upstream receptor tyrosine kinases (RTKs), RAS, RAF, or PI3K or loss of PTEN [54-57]. Similarly, acquisition of resistance to DNA damaging cytotoxic drugs can occur by mutation or expression changes of any one of a group of DNA damage response pathway genes [58, 59]. Consideration of these factors in sum may have considerable value in improving prognostic and diagnostic accuracy. While RNA and DNA were not available for the specimens analyzed by TMA in this study, our results suggest the value of a prospective study integrating measurement of p^T356^RB1, CDKN2A loss, and CCND1 amplification or overexpression

Our study has several limitations: particularly the retrospective nature, lack of cause-specific survival data, and the small sample size available for this analysis call for larger prospective studies in the future to validate our findings. It is also important to consider the inherent limitations of TCGA data. TCGA data is based on samples collected and processed at different institutions; molecular characterization is performed at different centers; and varying degrees of clinical annotation for samples has been noted [60]. Furthermore, differences in acquisition platforms and data processing pipelines may be confounding factors. In spite of these limitations, our work presents substantial evidence that p^T356^RB1 could be exploited to evaluated activity of the CDK4/6-cyclin D axis and thus may serve as a valuable predictive biomarker.

As noted in the introduction, recent data also link functional RB1 to responsiveness to pharmacological CDK4/6 inhibition, adding interest to our findings and raising the possibility that p^T356^RB1 may have utility in stratifying SCCHN patients for clinical trials of CDK4/6 inhibition. This is of particular interest as high levels of CDKN2A (p16) protein, as seen in HPV-positive SCCHN, are associated with limited response to CDK4/6 inhibition in several tumor types [25, 30, 32, 34, 35, 61]. Maximal possible benefit of these inhibitors to patients will greatly depend on reliable biomarkers to guide patient selection. In sum, our findings suggest that p^T356^RB1 status can function as an important survival predictor in HPV-negative SCCHN and should be tested as a potential marker for selection of patients for clinical trials.

## PATIENTS AND METHODS

### Patients

Tumor samples were obtained from archival formalin-fixed paraffin-embedded FCCC pathology specimens, collected at the time of initial surgery between 1990 and 2007. Institutional Review Board-approved consent forms were signed prior to sample collection. Five TMAs were constructed with tumor cores represented in duplicate and a selection of normal controls. Clinical data were extracted from FCCC clinical databases in an anonymized fashion. A total of 94 HPV-negative (all oropharyngeal primary tumors with either positive or unknown status of p16 were excluded) surgical SCCHN specimens were analyzed (Table 1).

### Fluorescence immunohistochemistry, image acquisition and AQUA analysis

Immunohistochemistry was performed as previously described [41]. Tissue sections were blocked with Background Sniper (BS966, Biocare Medical). Antigen Retrieval was performed in Tris/EDTA pH 9 Buffer for 20 minutes (S2367, Dako). The sections were incubated overnight with the appropriate primary antibody: p^T356^RB1 (1:200, 2223-1, Epitomics), RB1 (1:200, #9309, Cell Signaling Technology), p^T202/Y204^ERK1/2 (1:100, #9101S, Cell Signaling Technology), N-terminal TP53 (1:200, M7001, DAKO), or Ki-67 (1:800, AC-0009, Epitomics), and pan-cytokeratin (Rabbit 1:400, Z0622, Dako or Mouse, 1:100, M3515, Dako; tumor mask) in Da Vinci Green antibody diluent (PD900, Biocare Medical) at 4°C overnight. Signals were intensified with Envision reagents (DAKO). Pan-cytokeratin primary antibody was probed with an Alexa Fluor 555 dye-labeled secondary antibody (Invitrogen). Primary antibody visualization was accomplished using a Cy-5-tyramide signal amplification system (TSA; AT705A, PerkinElmer). Tissue nuclei were stained using Prolong Gold mounting medium (P36931; Molecular Probes) containing 4,6-diamidino-2-phenylindole (DAPI). HistoRx PM-2000 (HistoRx) with AQUAsition software was used for automated image capture as previously described [41].

### Statistical analysis

Patients eligible for analysis had a valid read (defined as detectable staining intensity within the dynamic range of the AQUA acquisition software, in the absence of staining artifacts) for at least one of the assessed proteins (Table 1). Associations between each marker's expression level, and grade, stage and survival, were assessed after choosing the optimal cutpoint using Classification and Regression Trees (CART; Supplementary Table S2; [62]), fit was determined using the rpart procedure in R software (version 3.0.2). Survival curves were generated using the methods of Kaplan and Meier [63], and tested for significance using Log-Rank tests. We further assessed the relationship between overall survival and marker expression levels by performing multivariate analysis using Cox proportional hazards regression [64], adjusting for T-stage, N-stage, grade, gender, the patient's age, and the specimen's age. The relationships between markers and stage/grade were analyzed using Spearman's correlation [65]. Correlations were presented graphically using the corrplot procedure in R.

### Cell culture, siRNA and western blot

FaDu and SCC61 cells from the ATCC were cultured as recommended by the suppliers. Transfection of cells with siRNA was accomplished using DharmaFECT1 (GE Healthcare) at a dilution ratio of 1:100 with serum free media. Depletion of proteins was accomplished using siRNA SMARTpools (four combined siRNAs per target) from GE Healthcare/Dharmacon: RB1 (NM_000321; cat.# M-003296-03), TP53 (NM_000546; cat.# M-003329-03), ERK2 (NM_138957; cat.# M-003555-04), and ERK1 (NM_001109891; cat.# M-003592-03). Scramble siRNA control was purchased from GE Healthcare/Dharmacon. Cells were plated in six well plates with the siRNA transfection mixture. After 48 hours, cells were lysed using M-PER Mammalian Protein Extraction Reagent (Thermo Scientific; #78501) supplemented with protease/phosphatase inhibitor cocktail (Thermo Scientific; #1861282). Western blotting was performed using standard procedures and was developed using SuperSignal West Pico Stable Peroxidase and Luminol/Enhancer solutions (Thermo Scientific; #1856135 & #1856136). Primary antibodies used were the same as described above, plus anti-β-actin conjugated to horseradish peroxidase (HRP; ab49900) from Abcam. All primary antibodies were used at a dilution of 1:1000; except anti-β-actin, which was used at 1:50,000. Secondary anti-rabbit and anti-mouse HRP-conjugated antibodies from GE Healthcare were used at dilutions of 1:10,000.

### TCGA data analysis

243 HPV-negative SCCHN specimens from the TCGA set [Head and Neck Squamous Cell Carcinoma (TCGA, in revision); [66]] were analyzed using cBioPortal (http://www.cbioportal.org; [67, 68]). Datasets reporting mRNA expression (RNA Seq V2 RSEM) and mRNA expression z-scores (RNA Seq V2 RSEM), mutations, putative copy-number alterations from GISTIC, as well as protein/phosphoprotein levels (RPPA) were retrieved. For mRNA, fold expression over the average was calculated and the corresponding z-scores were used as input for cBioPortal analysis. Kaplan-Meier survival curves and maps indicating DNA/RNA status were generated using cBioPortal.

## SUPPLEMENTARY FIGURES AND TABLES


