# Supplementary material for: Phospho-T356RB1 predicts survival in HPV-negative squamous cell carcinoma of the head and neck
Source: Oncotarget. 2015 May 29;6(22):18863–74. doi: 10.18632/oncotarget.4321 (PMC4662460; doi:10.18632/oncotarget.4321)
Supplement: Supplementary file 1 [file oncotarget-06-18863-s001.pdf]

Phospho-^{T356}RB1 predicts survival in HPV-negative squamous cell carcinoma of the head and neck

Supplementary Materials

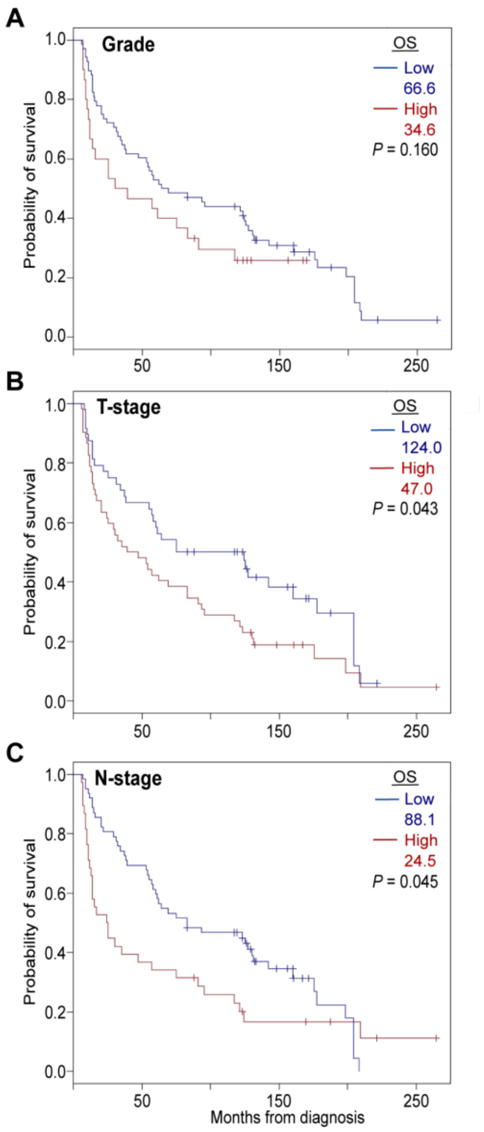

Supplementary Figure S1: Kaplan-Meier survival analysis for tumor grade and stage. (**A**) Low grade = well differentiated and moderately differentiated, high grade = poorly differentiated and undifferentiated, (**B**) low T-stage = 1, 1A, 2 and 2B, high T-stage = 3, 3A, 4, 4A and 4B, (**C**) low N-stage = 0 and 1, high N-stage = 2.

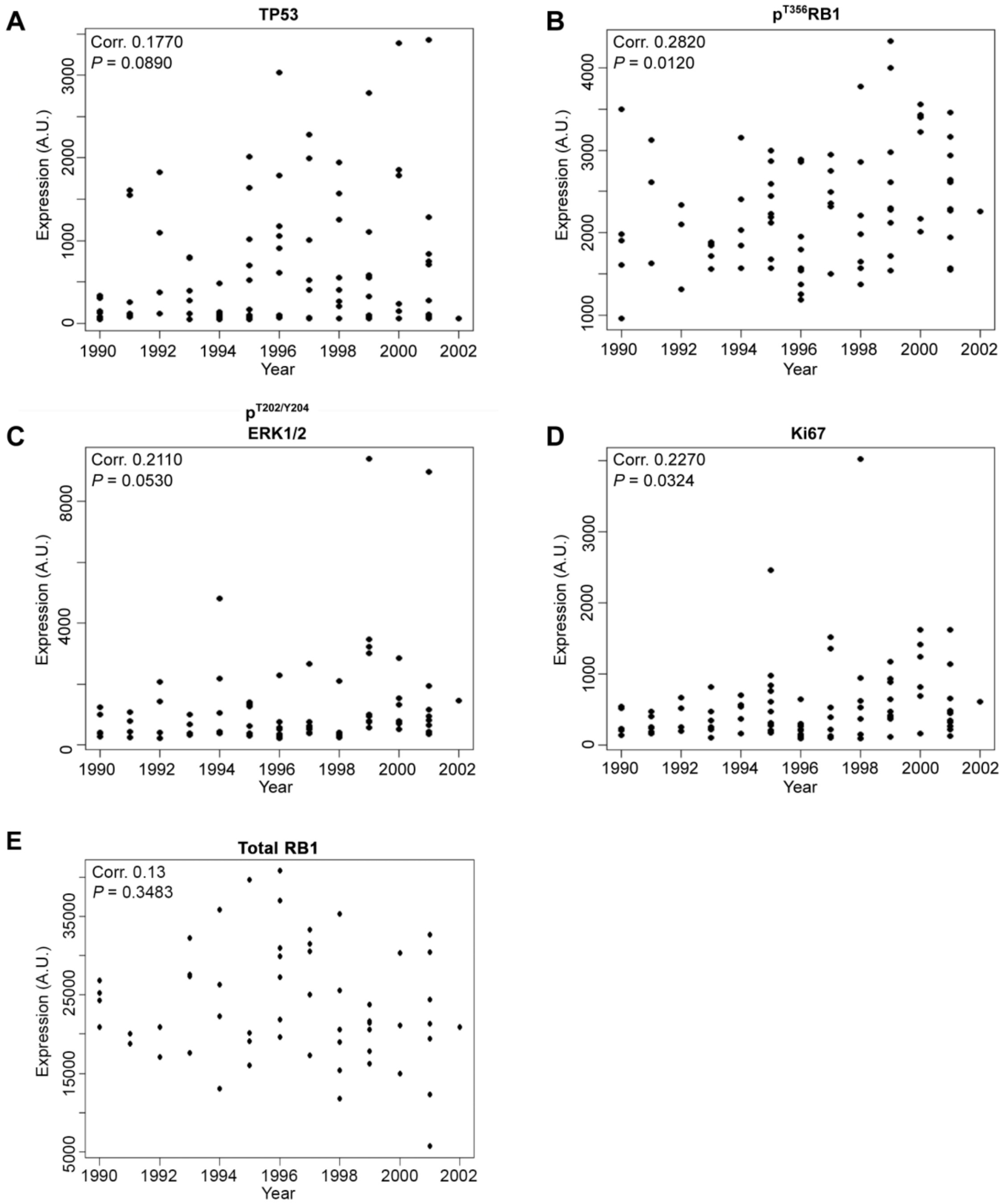

Supplementary Figure S2: Correlation between sample collection year and marker expression intensity. Corr. = correlation coefficient, A.U. = arbitrary unites.

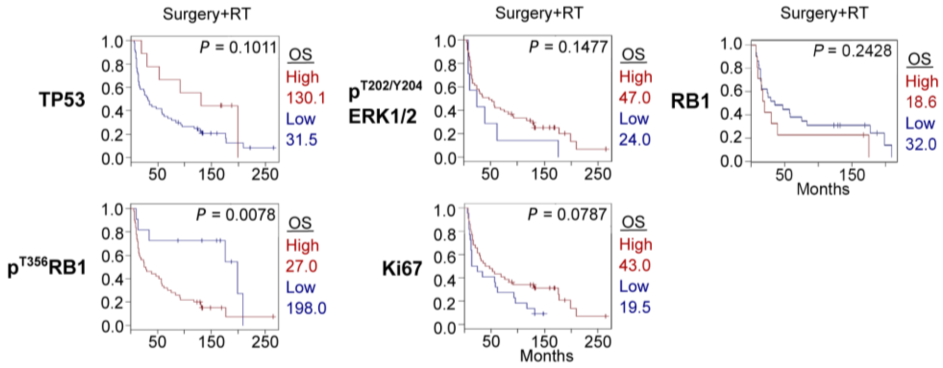

Supplementary Figure S3: Kaplan-Meier survival analysis for high and low expression levels of TP53, RB1, p^{T356}RB1, $p^{T202/Y204}$ERK1/2, and Ki67. Patients treated with surgery and radiation therapy were included. OS = overall survival.

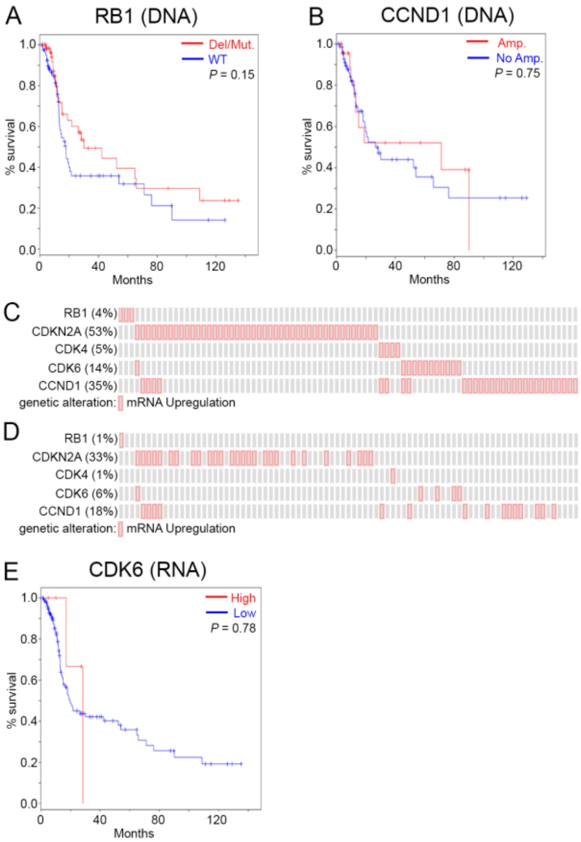

Supplementary Figure S4: Genomic and transcriptomic TCGA data analysis. (**A**) Kaplan-Meier survival curves for RB1 Del./Mut. (Shallow Deletions; Figure 5A) versus WT, (**B**) CCND1 amplification versus no amplification (Amp. Vs. No Amp.), (**C**) mRNA expression alterations with z > 2-fold and (**D**) z > 3-fold for RB1, CDK4, CDK6, CDKN2A (p16) and CCND1 in a set of 243 HPV-negative SCCHN TCGA specimens, (**E**) Kaplan-Meier survival curve for CDK6 mRNA expression differences. Mut. = mutation; Del. = deletion; WT = wild type; Amp. = amplification.

Supplementary Table S1: Adjusted survival analysis.

	All[a]				*Surgery + RT*[b]		
**Adjusted Survival**	**HR**[c]	**95% CI**[d]	***P*-value**		**HR**	**95% CI**	***P*-value**
**TB53**	0.4827	0.2321 - 1.0041	0.0513		0.40196	0.1609-1.004	0.051
low T-stage	0.6463	0.3981 – 1.0493	0.0775		0.76291	0.4106-1.418	0.392
low N-stage	0.5635	0.3259 – 0.9742	0.04		0.654	0.3543-1.207	0.175
low grade	0.6138	0.34 – 1.108	0.1053		0.63644	0.3375-1.2	0.163
gender (male)	0.7646	0.4597 – 1.2717	0.3011		0.86314	0.4637-1.607	0.643
age 65 and over	1.5198	0.9178 – 2.5165	0.1038		1.39862	0.7698-2.541	0.271
year 1994-1997[e]	1.2305	0.6229 – 2.4308	0.5504		1.08592	0.4993-2.362	0.835
year 1998-2002	0.8202	0.3932 – 1.7106	0.5971		0.69577	0.3188-1.519	0.362
**Ki67**	0.52217	0.2901-0.94	0.0303		0.5242	0.2807-0.9789	0.0427
low T-stage	0.74874	0.437-1.283	0.2923		0.882	0.4662-1.667	0.6991
low N-stage	0.56051	0.3194-0.984	0.0438		0.6688	0.3537-1.2643	0.2157
low grade	0.68727	0.3717-1.271	0.2318		0.6965	0.3536-1.372	0.2958
gender (male)	0.69271	0.407-1.179	0.1759		0.7357	0.386-1.402	0.3509
age 65 and over	1.3698	0.8185-2.292	0.231		1.135	0.6264-2.0566	0.6762
year 1994-1997	0.93583	0.4689-1.868	0.8508		0.7197	0.3255-1.5912	0.4165
year 1998-2002	0.77525	0.3627-1.657	0.5113		0.6904	0.3069-1.553	0.3705
**p^{T356}RB1**	2.9899	1.2188-7.3343	0.0167		3.279064	1.3194-8.149	0.0106
low T-stage	0.5788	0.316-1.0602	0.0766		0.845095	0.4185-1.706	0.6388
low N-stage	0.4602	0.2465-0.8591	0.0148		0.575105	0.297-1.114	0.1009
low grade	0.7744	0.4066-1.4749	0.4366		0.938944	0.4653-1.895	0.8604
gender (male)	0.787	0.4375-1.4154	0.4238		1.000987	0.5069-1.977	0.9977
age 65 and over	1.3936	0.7942-2.4452	0.2474		1.213837	0.6566-2.244	0.5365
year 1994-1997	1.1624	0.5233-2.5818	0.7118		1.111097	0.4782-2.582	0.8065
year 1998-2002	0.8411	0.3691-1.9167	0.6806		0.753286	0.3215-1.765	0.5143
**RB1**	1.4088	0.6512-3.048	0.3840				
low T-stage	0.6424	0.3116-1.325	0.2307				
low N-stage	0.5547	0.2833-1.086	0.0856				
low grade	0.6699	0.3262-1.376	0.2752				
gender (male)	0.8598	0.4335-1.706	0.6657				
age 65 and over	1.0859	0.553-2.133	0.8108				
year 1994-1997	1.1712	0.4719-2.907	0.7334				
year 1998-2002	1.0678	0.4137-2.756	0.8922				
**p$^{T202/Y204}$ERK1/2**	0.3874	0.149-1.0072	0.0517		0.42777	0.1588-1.152	0.093
low T-stage	0.7263	0.412-1.2804	0.2689		0.84308	0.4255-1.671	0.625
low N-stage	0.5181	0.2982-0.9002	0.0197		0.59372	0.3124-1.129	0.112
low grade	0.6899	0.3792-1.2551	0.2241		0.73463	0.3809-1.417	0.357
gender (male)	0.8909	0.4988-1.5914	0.6964		0.97299	0.4863-1.947	0.938
age 65 and over	1.5697	0.9057-2.7204	0.1081		1.28752	0.6847-2.421	0.433
year 1994-1997	0.8533	0.4161-1.75	0.6651		0.75297	0.3371-1.682	0.489
year 1998-2002	0.6749	0.32-1.4232	0.3016		0.60194	0.2686-1.349	0.218

[a]includes patients who received surgery only and patients who received surgery plus radiation therapy; [b]RT= radiation therapy; [c]HR = hazard ratio; [d]CI = confidence interval; [e]sample collection dates.

Supplementary Table S2: CART cutpoints for each marker and the number of samples in each category.

Group	Gene	P-value (OS)[a]	CART[b] found cutpoint	Best cutpoint	N[c] (total)	N above cutoff
All[d]	TP53	0.091629	TRUE	1587.122	94	14
Surgery+RT[e]	TP53	0.101093	TRUE	1783.751	69	9
All	RB1	0.111085	TRUE	30119.43	55	13
Surgery+RT	RB1	0.243766	TRUE	30119.43	38	10
All	p^{T356}RB1	0.02952	TRUE	1561.723	79	68
Surgery+RT	p^{T356}RB1	0.007779	TRUE	1561.723	61	50
All	Ki67	0.008236	TRUE	202.0592	89	69
Surgery+RT	Ki67	0.07868	TRUE	213.9365	66	44
All	$p^{T202/Y204}$ERK1/2	0.055768	TRUE	293.366	85	78
Surgery+RT	$p^{T202/Y204}$ERK1/2	0.147742	TRUE	295.3632	64	57

[a]OS = overall survival; [b]CART = Classificiation and Regression Trees; [c]N = number of samples; [d]includes patients who received surgery only and patients who received surgery plus radiation therapy; [e]RT = radiation therapy.
